# Princess Louise at Paddington Children's Hospital

**Published:** 1911-11-25

**Authors:** 


### Princess Louise at Paddington Children's Hospital.

HER Royal Highness the Princess Louise Duchess of Argyll, visited, on Thursday last week, the Paddington Green Children's Hospital, in order to declare open the new Out-Patient Department, the plans of which appeared in THE HOSPITAL last week. Her Royal Highness was received by Mr. Douglas Owen, Chairman of the Committee, Mr. George Hanbury, Treasurer, and the members of the Hospital staff. Mr. Douglas Owen gave a brief history of the circumstances which had led up to the present important extension. "Thirty years ago—in 1881—it was decided at a small meeting presided over by Mr. George Hanbury, to appeal for funds to replace the North-West London Free Dispensary, near Lisson Grove, by a Cottage Hospital for Children. Two or three of us on the present Hospital Committee were co-operators with Mr. Hanbury. Two newly erected houses on the present site were purchased, and in 1883 they were opened as a Children's Hospital with twenty-three beds. The Hospital ought to have been called—but for the refusal of Mr. Hanbury—the George Hanbury Hospital for Sick Children. We called it instead the Paddington Green Children's Hospital. Mr. Hanbury has, however, always been its treasurer, and until 1904 was Chairman of the Committee. In that year, at the request of Mr. Hanbury and the Committee, I relieved him of the duties of chairmanship. It is wonderful to me how we ever managed to obtain the £13,000 required to build and equip the present building, which in 1895, provided with forty-six cots, was graciously opened by H.R.H. the late Princess Mary Adelaide, Duchess of Teck. Three years later the Duchess of York (now her Gracious Majesty the Queen) visited the Hospital, and named the present 'Mary Adelaide' Ward. But for some years it has been increasingly impressed on the committee that the out-patient accommodation provided in 1888 had, owing to the rapid growth of the Hospital's population area, become quite inadequate. So impressed were the committee of King Edward's Hospital Fund with the need of the extension that they promised first £1,000 and later £1,250 in addition towards the cost. The promise was, however, conditional on the committee's approval of our plans. To our dismay the committee called for a modification or, rather, extension involving a further outlay of about £2,000 in site purchase and building, and as the result we are now £2,000 to the bad. On the other hand we recognise cheerfully that the action of the King's Fund has been beneficial. The new out-patient extension is splendid, and the Hospital Committee, as well as the medical and surgical staff, regard it with pride. As to the £2,000 debt, we have met with such kindness and generosity in the past that it would be ungrateful to think that it will long be allowed to remain a burden on the hospital. It is only by legacies that we are enabled to pay our way at all. In the present extension we count ourselves peculiarly fortunate. Fortunate most and first of all in our architect, Mr. Alan Munby, whose ability, resourcefulness, and pains have given us an accommodation quite beyond our hopes. The contractors also, Messrs. Henwood, have left us in their debt—old friends of the hospital, having secured the contract by the lowest tender, they made its carrying out a matter of personal care. And finally, and most notably, your Royal Highness, with a graciousness for which we are most grateful, has come to us to declare the building open."

Her Royal Highness then placed a gold key in the lock of an electric box on the table before her, and on turning it folding doors flew open, a tablet on the wall was uncovered, and the Princess, amid loud applause, declared the new building to be opened.

Sir Frederick Milner and Sir Henry Seton-Karr having moved a vote of thanks to the Princess for her gracious kindness in attending, the Duke of Argyll made an interesting speech, endorsing the importance of societies for the education of mothers.

A large number of purses were then presented by children to her Royal Highness, who was then conducted over the new building by the Chairman, the architect, Mr. Alan Munby, the secretary, Mr. W. H. Pearce, and the matron, Miss McGregor, and the senior members of the staff.

The Chairman explained that it was hoped that the friends of the Hospital would in course of time provide picture tile panels for the walls of the out-patient waiting room, as an interest and distraction to the children waiting their turn. Four of these panels had, in fact, already been promised, and were to be supplied by Messrs. Craven Dunhill and Co. The Princess then visited the new museum to inspect the display of articles contributed by the Ladies' Work Association, of which Mrs. Douglas Owen is the president, and the committee of which was presented to her Royal Highness.

